# Short-term outcomes after hospital discharge in patients admitted with heart failure in Abeokuta, Nigeria: Data from the Abeokuta Heart Failure Registry

**DOI:** 10.5830/CVJA-2014-040

**Published:** 2014

**Authors:** Okechukwu S Ogah, Ayodele O Falase, Simon Stewart, Karen Sliwa, Joshua O Akinyemi, Gail D Adegbite, Albert A Alabi, Amina Durodola, Akinlolu A Ajani

**Affiliations:** Division of Cardiology, Department of Medicine, University College Hospital, Ibadan, Nigeria; Soweto Cardiovascular Research Unit, Faculty of Health Sciences, University of the Witwatersrand, Johannesburg, South Africa; Division of Cardiology, Department of Medicine, University College Hospital, Ibadan, Nigeria; NHMRC Centre of Research Excellence to Reduce, Inequality in Heart Disease Baker IDI Heart and Diabetes Institute, Melbourne, Australia; NHMRC Centre of Research Excellence to Reduce, Inequality in Heart Disease Baker IDI Heart and Diabetes Institute, Melbourne, Australia; Department of Epidemiology and Medical Statistics, College of Medicine, University of Ibadan, Nigeria; Hatter Institute for Cardiovascular Research in Africa and IIDMM, Department of Medicine, Faculty of Health Sciences, University of Cape Town, South Africa; Department of Epidemiology and Medical Statistics, College of Medicine, University of Ibadan, Nigeria; Department of Medicine, Sacred Heart Hospital, Lantoro, Abeokuta, Nigeria; Department of Medicine, Sacred Heart Hospital, Lantoro, Abeokuta, Nigeria; Department of Medicine, Federal Medical Centre, Abeokuta, Nigeria; Department of Medicine, Federal Medical Centre, Abeokuta, Nigeria

**Keywords:** heart failure, mortality, outcome, Abeokuta, Nigeria

## Abstract

**Background:**

Compared to other regions of the world, there is a paucity of data on the short-term outcome of acute heart failure (AHF) in Africa’s most populous country, Nigeria. We examined the six-month outcomes (including case fatalities) in 285 of 309 AHF subjects admitted with HF to a tertiary hospital in Abeokuta, Nigeria.

**Methods:**

The study cohort of 285 subjects comprised 150 men (52.6%) and 135 women (47.4%) with a mean age of 56.3 ± 15.6 years and the majority in NYHA class III (75%).

**Results:**

There were a number of differences according to the subject’s gender; men being older and more likely to present with hypertensive heart disease (with greater left ventricular mass) while also having greater systolic dysfunction. Mean length of stay was 10.5 ± 5.9 days. Mean follow up was 205 days, with 23 deaths and 20 lost to follow up. At 30 days, 4.2% (95% CI: 2.4–7.3%) had died and by 180 days this had increased to 7.5% (95% CI: 4.7–11.2%); with those subjects with pericardial disease demonstrating the highest initial mortality rate. Over the same period, 13.9% of the cohort was re-admitted at least once.

**Conclusions:**

The characteristics of this AHF cohort in Nigeria were different from those reported in high-income countries. Cases were relatively younger and presented with non-ischaemic aetiological risk factors for HF, especially hypertensive heart disease. Moreover, mortality and re-admission rates were relatively lower, suggesting region-specific strategies are required to improve health outcomes.

## Abstract

Heart failure (HF) has emerged as a global epidemic in at-risk populations, including those living in high-income countries and, as recently described, in low- to middle-income regions of the world, such as sub-Saharan Africa.^1^1-4 While there are well-established HF registries to capture both the characteristics and health outcomes among those hospitalised with AHF in Europe,^5^,^6^ North America,^7^,^8^ and the Asia–Pacific region,^3^,^9^,^10^ there are few reports from sub-Saharan Africa.^11^ This includes Nigeria (the most populous country in the region), where HF has emerged as a potentially large public health problem.^1^

Although there have been many therapeutic gains in the management of chronic HF,^12^ leading to improved overall survival rates,^13^ there has been very little parallel success (pending further evaluation of the recently reported RELAX trial^14^ with regard to AHF). This is particularly important when one considers the high proportion of patients who still require hospitalisation for acute HF, and associated high levels of in-patient case fatality and poor short- to medium-term health outcomes.

Given the paucity of data describing health outcomes in unselected patients hospitalised with AHF in Nigeria (and indeed the wider sub-Saharan Africa), we examined short- (30 days) to medium-term outcomes (180 days) in consecutive subjects with AHF recruited into the Abeokuta HF registry over a period of six months. Standardised data collected via the registry were used to both describe the baseline characteristics of the cohort and identify correlates of mortality during the six-month follow up.

## Methods

The Abeokuta HF registry was a hospital-based, single-centre, prospective, observational study that consecutively recruited 285 subjects with *de novo* AHF and 24 cases of decompensated HF (acute-on-chronic HF), all admitted during the period 1 January 2009 to 31 December 2010. The 24 cases of decompensated HF were excluded from the final analysis.

The main objective of the registry was to characterise the current profile of HF in the community. It was also aimed at determining the mode of care as well as intra-hospital and six-month outcomes.

Clinical information relating to the socio-demography, medical history, signs and symptoms, medications, results of laboratory investigations, including 12-lead ECG and echocardiography, were collected. A standardised case report form was used for data collection. Home addresses and telephone contacts of the subjects as well as their next of kin were also recorded.

Subjects were weighed without shoes and in light clothing using a standard beam balance. An anthropometric plane was used for height measurement to the nearest centimetre. Body mass index (BMI) was calculated using the standard formula. Blood pressure measurements were done according to international guidelines,^15^ with the use of a mercury sphygmomanometer (Accousson, London).

We defined anaemia as haematocrit of less than 10 g/dl. The modification of diet in renal disease (MDRD) formula was used for the estimation of glomerular filtration rate (GFR).^16^ An estimated GFR (eGFR) of less than 60 ml/min/1.73 m^2^ was the criterion used for defining renal dysfunction.^4^

A clinical diagnosis of HF was based on the Framingham criteria.^17^ Using the recent guidelines of the European Society of Cardiology,^18^ subjects were categorised into *de novo* presentation, as well as recurrent presentation of typically decompensated HF (i.e. acute-on-chronic HF).

Standard 12-lead resting ECGs were recorded for each patient using a Schiller ECG machine (Schiller AG, Switzerland). All the 12-lead resting ECGs were performed by trained nurses/technicians and analysed by a reviewer who was blinded to the clinical data of the patients.

Echocardiography was performed on the subjects with the use of an Aloka SSD – 4000 echocardiography (Aloka Co Ltd, Tokyo, Japan). Standard views and two-dimensional guided M-mode measurements were obtained according to international guidelines. Aortic root and left atrial diameter, left ventricular (LV) internal dimensions and wall thicknesses were obtained according to the American Society of Echocardiography (ASE) criteria. Measurements were obtained in up to three cycles and averaged. One experienced cardiologist (OSO) performed all the procedures.

In our laboratory, the intra-observer concordance correlation coefficient and measurement errors have been reported.^19^ The Devereux and Recheck formula was used for LV mass calculation.^20^ Increased relative wall thickness (RWT) was defined as RWT > 0.43.^21^

Impaired LV systolic function was defined as LV ejection fraction of < 50%. Transmitral flow velocities, deceleration time and isovolumic relation time were obtained using standard methods.^22^ Tissue Doppler imaging (TDI) was applied only to identify true pseudo-normalised filling pattern.

**Fig. 1.**
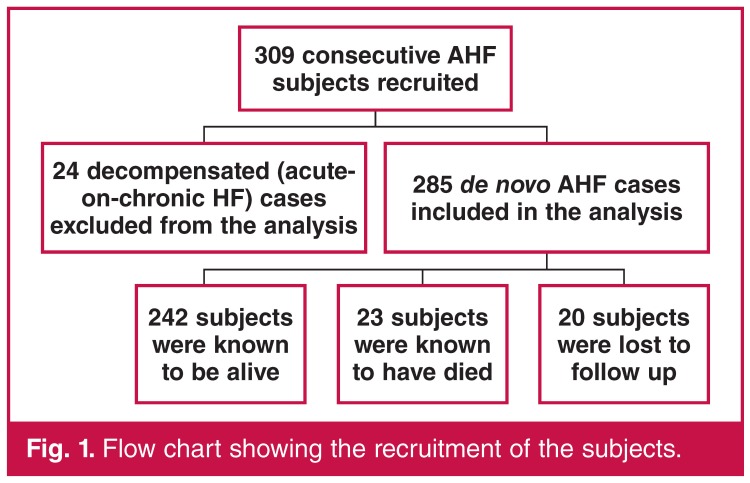
Flow chart showing the recruitment of the subjects.

The cohort was prospectively followed up for six months. The mean follow-up period was 205 days. Subjects were contacted via clinic visits or telephone calls at one, three and six months. Follow-up data included their wellbeing, medications, history of rehospitalisation and deaths (from next of kin). In addition to patient or relative telephone interviews, where necessary, referring physicians were contacted for additional information. Fig. 1 is a flow chart showing the recruitment and follow up of the study cohort.

We examined (1) length of hospital stay (LoS), (2) Survival status on discharge (dead or alive), (3) short-term case fatality/re-admission (30 days), (4) medium-term case fatality (within 180 days), (5) rehospitalisation status (within 180 days), and (6) event-free survival from re-admission or death.

The study was reviewed and approved by the institution’s ethics review board. All the subjects gave informed consent and the study was carried out in accordance with the Declaration of Helsinki.^23^

## Statistical analysis

Data were entered into EpiData software. The EpiData association (att. Jens Lauritsen, Enghavevej 34, DK5230 Odense M, Denmark) was used for data entry, while SPSS version 15 and Stata version 11.1 were used for data cleaning and analysis. Continuous variables are presented as means and standard deviations (SDs), or medians with their 25th and 75th percentiles when the distribution of the data did not follow Gaussian distribution.

Categorical variables are displayed as frequencies and proportions. Group comparison was done with the Student’s *t*-test, and chi-square statistics were used for comparison of categorical variables. Survival function estimates were performed using the Kaplan–Meier method and the difference was tested using the log-rank test. The follow up was censored at six months post admission.

Predictors of survival were determined using univariate regression analyses. Thereafter multiple logistic regression analysis was performed to identify independent predictors of survivals (*p* < 0.1 used for selection of variables).

Results are expressed as odds ratio (OR) with their 95% confidence intervals (95% CI). Odds ratios that were significantly greater than 1.00 implied that subjects with that attribute had higher risks of death compared to subjects who did not. A *p*-value of < 0.05 was taken as significant.

## Results

Overall, there were 150 men (52.6%) and 135 (47.4%) women (Table 1). The mean age was 56.3 ± 15.6 years (57.0 ± 13.6 and 55.4 ± 17.6 years for men and women, respectively) with 46% aged ≥ 60 years. Around one-third had no formal education, two-thirds were married and most (75.8%) were urban residents. The majority of the subjects were in NHYA class III (75.4%).

**Table 1:** Demographic and clinical profile characteristics of the cohort.

*Variable*	*All (n = 285)*	*Men (n = 150)*	*Women (n = 135)*	*p-value*
Socio-demographic variables				
Age (years)	60.0 ± 13.2	57.0 (13.6)	55.6 (17.3)	0.382
Age > 60 years (%)	46.3	48.7	43.7	0.425
No education	98 (34.4)	39 (26.0)	59 (43.7)	0.028
Married (%)	156 (67.8)	92 (73.0)	64 (61.0)	0.014
Unemployed	7 (2.3)	1 (0.6)	6 (4.2)	0.007
Urban residence	216 (75.8)	113 (75.3)	103 (76.5)	0.389
Risk factors and co-morbidities				
Never smoked cigarettes	233 (81.8)	103 (68.7)	103 (96.3)	< 0.001
Current alcohol use	17 (6.0)	14 (9.3)	3 (2.2)	< 0.001
Diabetes mellitus	37 (13.0)	19 (12.7)	18 (13.3)	0.735
Hypertension	232 (81.4)	128 (85.3)	134 (77)	0.103
COPD	20 (7.0)	11 (7.3)	9 (6.7)	0.923
Family history of heart disease	25 (8.8)	9 (6.0)	16 (11.9)	0.240
Clinical/laboratory parameters				
NYHA class				
Class II	24 (8.4)	16 (10.7)	8 (5.9)	0.212
Class III	215 (75.4)	107 (71.3)	108 (80.0)	
Class IV	46 (16.1)	27 (18.0)	19 (14.1)	
BMI (kg/m^2^)	25.2 ± 5.7	24.1 (5.0)	23.7 (5.5)	0.527
Systolic BP (mmHg)	131.9 ± 25.1	137.9 (30.0)	133.3 (27.9)	0.253
Diastolic BP (mmHg)	85.4 ± 15.9	89.0 (19.6)	85.3 (17.1)	0.156
Pulse pressure (mmHg)	46.5 ± 15.7	49.0 (19.0)	47.7 (16.6)	0.527
Respiratory rate (cycles/min)	30.2 ± 6.5	28.5 ± 6.4	27.9 ± 6.7	0.541
Pulse rate (bpm)	95.9 ± 16.7	96.2 ± 18.2	96.3 ± 17.8	0.527
Packed cell volume (%)	35.9 ± 7.8	37.5 ± 7.2	36.8 ± 7.7	0.541
Total white blood cell count (× 10^9^ cells/l)	6.4 ± 2.9	7.3 ± 3.7	7.4 ± 3.8	0.933
Serum sodium (mmol/l)	136.5 ± 6.4	135.9 ± 6.7	136.3 ± 6.1	0.134
Serum potassium (mmol/l)	3.7 ± 0.8	3.7 ± 0.8	3.6 ± 0.8	0.461
Total cholesterol (mg/dl)	162.5 ± 53.3	157.7 ± 84.0	181.2 ± 64.6	0.213
Serum glucose (mg/dl)	111.7 ± 53.2	115.6 ± 50.6	114.0 ± 58.5	0.845
Serum urea (mg/dl)*	38.5 ± 30.0	50.5 ± 51.4	36.1 ± 29.7	0.020
Serum creatinine (mg/dl)*	1.8 ± 0.4	1.7 ± 2.5	1.2 ± 1.4	0.093

COPD = chronic obstructive pulmonary disease.

The women were more likely not to have had formal education (43.7 vs 26.0%, *p* = 0.029), more likely not to be a smoker (96.3 vs 68.7%, *p* < 0.001), and less likely to be a current alcohol user (2.2 vs 9.3%, *p* < 0.001). Alternatively, men had higher rates of hypertension (85.3 vs 77.0%) and chronic obstructive pulmonary disease (COPD) (7.3 vs 6.7%).

Table 2 shows the laboratory profile, aetiological risk factors and discharge medications. Serum urea and creatinine concentrations were significantly higher in men than women.

**Table 2:** Aetiology of HF and discharge medications in the 285 subjects.

*Variable*	*All (n = 285)*	*Men (n = 150)*	*Women (n = 135*
Aetiology of HF, *n* (%)			
Hypertension	216 (75.8)	119 (79.3)	97 (71.9)
Dilated cardiomyopathy	24 (8.4)	16 (10.7)	8 (5.9)
Cor pulmonale	16 (5.6)	9 (6.0)	7 (5.2)
Pericardial diseases	9 (3.2)	1 (0.7)	8 (5.9)
Rheumatic heart disease	7 (2.5)	4 (2.7)	3 (2.2)
Peripartum cardiomyopathy	6 (2.1)	0 (0.0)	6 (4.4)
Thyroid heart disease	3 (1.1)	0 (0.6)	3 (2.2)
Ischaemic heart disease	1 (0.4)	1 (0.7)	0 (0.0)
Adult congenital heart disease	1 (0.4)	0 (0.0)	1 (0.7)
Endomyocardial fibrosis	2 (6.7)	0 (0.0)	2 (0.7)
Type of heart failure			
Systolic heart failure (%)	66.4	71.4	60.9
Heart failure with normal EF (%)	33.6	28.6	39.1
Medications, *n* (%)			
Loop diuretics	249 (87.4)	132 (88.0)	117 (86.7)
Digoxin	219 (76.8)	114 (76.0)	105 (77.8)
ACE inhibitors/ARBs	281 (98.6)	148 (98.7)	133 (98.5)
Beta-blockers	56 (19.6)	35 (23.3)	21 (15.6)
Spironolactone	247 (86.7)	133 (87.3)	116 (85.9)
Hydrallazine–isosorbide	33 (11.7)	19 (12.9)	14 (10.4)
Amiodarone	5 (1.8)	4 (2.7)	1 (0.7)

Except for peripartum cardiomyopathy (PPCM), the aetiological risk factors were similar in men and women. Hypertensive heart disease was found in 75.8% of patients, dilated cardiomyopathy in 8.4%, cor pulmonale in 5.6%, pericardial diseases in 3.2% and rheumatic heart disease in 2.5%. PPCM, thyroid heart disease, coronary artery disease and endomyocardial fibrosis were found in 2.1, 1.1, 0.4, 0.4 and 0.7% of patients, respectively.

The discharge medications were similar in men and women except for beta-blockers, which were prescribed more in men.

Table 3 depicts the 12-lead ECG and echocardiographic parameters according to gender. Men had significantly higher mean absolute QT intervals (374 ± 35.0 vs 348 ± 45.5 ms, *p* = 0.006), left atrial area (28.8 ± 8.8 vs 25.0 ± 6.4 cm^2^, *p* = 0.010), LV internal dimension in systole, as well as absolute and indexed LV mass (*p* = 0.001, 0.026 and 0.016, respectively). On the other, hand women had significantly higher ejection fractions (45.1 ± 20.1 vs 40.6 ± 23.6, *p* = 0.007).

**Table 3:** Twelve-lead ECG and echocardiographic profile according to gender.

*Variable*	*All (n = 285)*	*Men (n = 150)*	*Women (n = 135)*	p*-value*
Ventricular rate (bpm)	96.3 ± 22.5	94.3 ± 17.3	101.3 ± 21.8	0.110
QRS duration (ms)	116.0 ± 26.2	117.1 ± 24.5	107.8 ± 41.1	0.213
QT interval (ms)	350.7 ± 30.6	374.3 ± 35.0	348.8 ± 45.5	0.006
Corrected QT (ms)	442.0 ± 20.9	462.2 ± 38.2	447.6 ± 36.2	0.085
Atrial fibrillation (%)	13.3	16.7	9.6	0.337
Aortic root diameter (cm)	3.2 ± 0.6	3.26 ± 0.58	2.84 ± 0.38	< 0.001
Left atrial diameter (cm)	5.9 ± 0.8	4.75 ± 0.89	4.50 ± 0.85	0.176
Left atrial area (cm^2^)	30.15 ± 9.91	28.8 ± 9.0	24.7 ± 6.3	0.010
IVSD (cm)	1.18 ± 0.28	1.33 ± 0.39	1.23 ± 0.32	0.393
LVPWd (cm)	1.38 ± 0.35	1.19 ± 0.39	1.10 ± 0.35	0.116
LVIDd (cm)	5.52 ± 0.97	5.81 ± 1.61	5.16 ± 1.45	0.353
LVIDs (cm)	4.51 ± 1.57	4.80 ± 1.63	4.16 ± 1.43	0.001
Fractional shortening (%)	14.5 ± 2.97	17.77 ± 13.10	19.80 ± 12.21	0.060
Ejection fraction (%)	36.8 ± 6.53	40.57 ± 23.61	45.12 ± 20.11	0.007
E/A ratio	2.11 ± 1.55	2.14 ± 1.47	1.90 ± 1.25	0.199
DT (ms)	145.8 ± 59.2	144.2 ± 58.3	147.9 ± 60.5	0.480
IVRT (ms)	111.0 ± 34.3	114.9 ± 35.8	106.1 ± 32.1	0.127
LV mass (absolute)	449.0 ± 217.5	561.7 ± 106.6	233.0 ± 54.24	0.026
LV mass (indexed)	274.1 ± 117.5	336.4 ± 46.6	160.9 ± 16.1	0.016
Mitral regurgitation (%)	19.6	18.7	20.7	0.894
Tricuspid regurgitation (%)	15.1	12.7	17.8	0.459

IVSD = interventricular septal wall thickness in diastole, LVPWd = left ventricular posterior wall thickness in diastole, LVIDd = left ventricular internal diameter in diastole, LVIDs = left ventricular internal diameter in systole, DT = deceleration time, IVRT = isovolumic relaxation time.

The mean length of hospital stay was 10.5 ± 5.9 days, (11.0 ± 5.4 and 10.0 ± 6.3 days for women and men, respectively). Mortality rate at 30 days was 4.2% (95% CI: 2.4–7.3) for the whole cohort. It was 3.9% (95% CI: 1.7–8.5%) and 4.5% (95% CI: 2.1–9.3%) for men and women, respectively. At 180 days, the mortality rate was 7.3% (95% CI: 4.7–11.2%). This was 7.1% (95% CI: 3.8–12.7%) and 7.5% (95% CI: 3.9–14.0%) for men and women respectively.

**Fig. 2.**
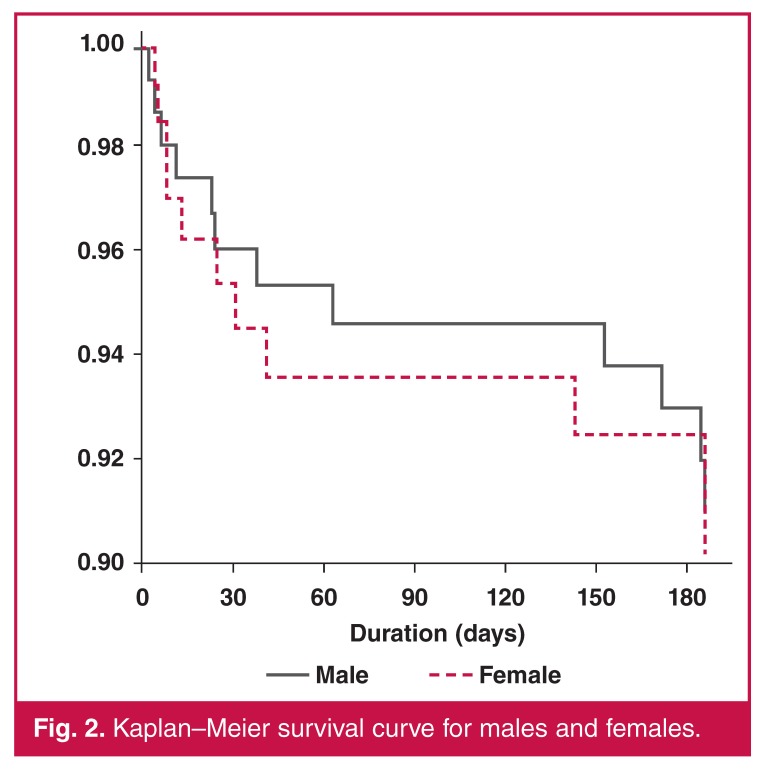
Kaplan–Meier survival curve for males and females.

**Fig. 3.**
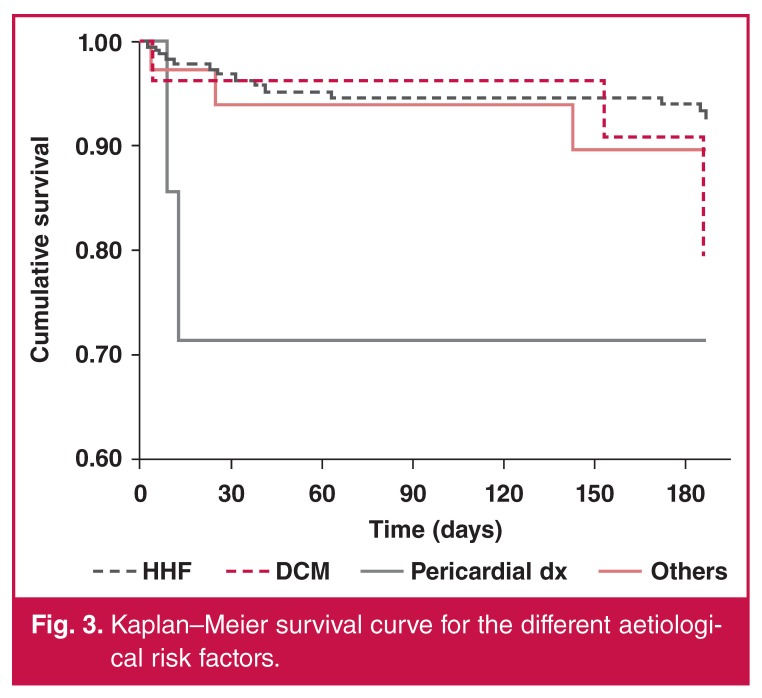
Kaplan–Meier survival curve for the different aetiological risk factors.

Patients with pericardial diseases had the highest early mortality rate. Hypertensive HF subjects had the best survival rates (Figs 1, 2, 3). At 180 days, 13.9% of the subjects were rehospitalised at least once (14.6% for women and 13.3% for men).

Table 4 shows the univariate correlates of survival in the cohort. Mortality was associated with female gender, being single, HF with normal ejection fraction, lower blood pressure, higher heart and respiratory rates, higher body temperature, anaemia, high creatinine levels and higher total white blood cell counts. Other factors included higher QRS duration and corrected QT interval, larger left atrial diameter and area, higher NYHA class and presence of tricuspid and mitral regurgitation. In a multiple regression analysis for predictors of mortality at 180 days, none of these variables reached statistical significance.

**Table 4:** Clinical and demographic predictors of outcome on univariate analysis (six-month survival).

*Variable*	*All (n = 285)*	*Alive (258)*	*Dead (23)*	*OR*	*95% CI*	*p-value*
Age (years)	57.3 ± 15.4	57.4 ± 14.0	57.2 ± 19.1	0.99	0.96–1.01	0.324
Female gender (%)	52.6	54.5	50	1.14	0.48–2.70	0.764
No education (%)	33.3	32.8	30.0	0.77	0.26–2.28	0.635
Not married (single) (%)	67.8	69.6	52.9	1.51	0.56–4.07	0.417
Body mass index	24.0 ± 5.4	23.7 ± 4.9	23.4 ± 3.6	0.97	0.87–1.08	0.580
Non-smoker (%)	81.8	82.3	85.0	1.51	0.43–5.34	0.521
Alcohol use (%)	6.0	5.6	5.0	0.79	0.32–1.95	0.609
Presence of diabetes (%)	13.0	13.1	10.0	0.65	0.14–2.92	0.574
Respiratory rate (bpm)	28.3 ± 6.2	28.0 ± 6.6	29.2 ± 5.3	1.02	0.96–1.08	0.639
Heart rate (bpm)	95.5 ± 17.1	95.0 ± 17.4	100.5 ± 15.9	1.00	0.97–1.03	0.846
SBP (mmHg)	136.1 ± 29.4	137.3 ± 27.7	122.5 ± 20.0	0.98	0.96–6.99	0.017
DBP (mmHg)	87.1 ± 29.4	88.6 ± 18.7	80.0 ± 13.4	0.98	0.95–1.00	0.085
Pulse pressure > 30 mmHg (%)	3.3	2.1	5.0	0.42	0.16–1.10	0.078
NYHA (III, IV) (%)	91.5	90.4	95.0	4.03	1.53–10.65	0.005
Serum sodium (mmol/l)	136.9 ± 4.6	136.0 ± 6.4	137.2 ± 7.4	1.03	0.96–1.11	0.428
Serum potassium (mmol/l)	3.7 ± 0.5	3.6 ± 0.7	4.0 ± 1.0	1.64	0.72–3.75	0.243
Blood glucose (mg/dl)	112.3 ± 56.0	117.0 ± 58.5	111.8 ± 58.5	1.00	0.99–1.01	0.501
Packed cell volume (%)	41.0 ± 7.6	37.6 ± 7.0	32.2 ± 8.4	0.92	0.86–0.97	0.004
Total white blood cell count	6.8 ± 3.1	6.9 ± 3.4	9.2 ± 5.1	1.13	1.02–1.25	0.024
Serum creatinine (mg/dl)	0.8 ± 0.3	1.2 ± 1.0	2.1 ± 2.5	1.38	1.04–1.83	0.024
QRS duration (ms)	107.1 ± 9.4	110.3 ± 29.5	110.9 ± 32.2	1.01	0.99–1.03	0.171
Corrected QT (ms)	439.4 ± 40.9	449.3 ± 34.4	457.3 ± 34.6	1.01	0.99–1.04	0.173
Atrial fibrillation (%)	13.3	14.6	20.0	1.14	0.36–3.55	0.827
E/A ratio	2.2 ± 1.0	2.1 ± 1.3	2.7 ± 1.6	1.40	0.99–1.97	0.060
Left atrial area (cm^2^)	26.2 ± 6.7	26.8 ± 7.5	34.2 ± 12.1	1.11	1.01–1.21	0.025
Left atrial diameter (cm)	4.8 ± 0.9	4.6 ± 0.9	5.0 ± 1.1	1.56	0.94–2.60	0.084
LVID (cm)	5.47 ± 1.55	5.6 ± 1.5	5.7 ± 1.2	1.11	0.74–1.67	0.614
HF with systolic dysfunction	66.4	67.5	70.6	0.66	0.27–1.59	0.356
MR (yes) (%)	19.6	20.2%	25.0	1.34	0.50–3.60	0.562
TR (yes) (%)	15.1	13.1%	35.0	2.64	1.00–6.95	0.050

SBP = systolic blood pressure, DBP = diastolic blood pressure, LVID = left ventricular internal diameter, MR = mitral regurgitation, TR = tricuspid regurgitation.

## Discussion

This is the first detailed study of the clinical profile and shortor medium-term outcome of AHF cases in southern Nigeria. Similar to our earlier observation,^24^ AHF in our community predominantly affects younger and middle-aged individuals who are in the prime of their lives. Hypertensive heart disease and other non-ischaemic aetiology contribute to over 90% of the cases.

The majority of our subjects presented with *de novo* acute HF. Our findings with the use of some disease-modifying agents such as angiotensin converting enzyme (ACE) inhibitors or angiotensin II receptor blockers (ARBs), aldosterone antagonists (except for beta-blockers and hydralazine–isosorbide combination) are remarkably similar to findings in many other parts of the world.^6^,^8^ Mortality rates in the short and medium term are relatively low, and higher in women than men.

Our findings of relatively young age at presentation for AHF is similar to reports from many parts of Africa.^1^,^4^,^25^ AHF patients on the continent are about 20 years younger than similar patients in high-income countries.^6^-^9^ This implies that HF afflicts our population in their productive years, with attendant economic loss to the society and greater disability-adjusted life years.

The comparable or even lower short- or medium-term mortality rate of HF in our cohort compared to findings in high-income countries is an important observation from this study.^7^,^8^ Mortality rates in our study were 4.2% (95% CI: 2.4–7.3%) and 7.3% (95% CI: 14.7–11.2%) at 30 days and 180 days, respectively.

Unlike findings in high-income countries,^26^,^27^ we noted that age was not associated with poorer outcome in our cohorts. Our finding of a better prognosis in obese individuals is similar to that of other researchers.^27^,^28^ In the Framingham study, high BMI was associated with a better prognosis (HR for mortality per one SD: 0.88, 95% CI: 0.75–1.04 for men, and 0.86, 95% CI: 0.72–1.03 for women). This may also be consistent with the ‘obesity paradox’ in HF.^29^-^31^ Underweight in HF patients may be indicative of cardiac cachexia, and progression of HF and poor prognosis.

Lower blood pressure or pulse pressure was associated with a poorer outcome. This may reflect advanced HF and decreased stroke volume. This has been noted in previous studies.^26^,^32^

It is now well known that impaired renal function is an important predictor of all-cause mortality in HF.^33^-^35^ This is similar to the observation in our study. Patients with renal impairment often develop cardio-renal syndrome, which is caused by low cardiac output. These patients often develop multiple alterations at the vascular level, leading to endothelial dysfunction, coagulation abnormalities, insulin resistance, hyperhomocystinaemia and activation of the sympathetic nervous system, as well as the renin–angiotensin and aldosterone system. They are prone to unstable HF and susceptible to high catecholamine levels. Furthermore HF patients with renal dysfunction are also less likely to receive proven medications for HF.

Hyponatraemia and hypokalaemia were associated with a better prognosis in our study. This is contrary to most reports from the Western world, although in a Polish study, Biegus *et al.*^8^ reported that hypokalaemia was associated with a better outcome. This may be related to better response to diuretics in the survivors, leading to the electrolyte derangement. It may also be speculated that sodium may play a lesser role in the pathophysiology of HF in our setting.

We also observed that left atrial size, left atrial area, left ventricular size, higher E/A ratio and presence of mitral and tricuspid regurgitation were associated with poorer outcomes. This has been well recognised by earlier studies.^7^,^9^ Left atrial or ventricular size reflects left atrial or ventricular pressure and volume overload, and the severity and duration of increases in LV filling in response to cardiac functionl abnormality associated with HF.^36^

A plausible reason for the younger age at presentation for HF in our study and many parts of Africa may be related to the aetiology of the condition, which is conditions that present in young and middle age (for example rheumatic heart disease and cardiomyopathies). In addition, hypertension and related target-organ damage present at a younger age in Africans and people of African descent.

The dominance of de novo presentation of HF in our cohort may be related to poorer long-term outcome of HF in our setting, that is, few people are living with chronic HF. Another reason may be because of poor or inadequate health education. Most often patients do not keep to one health facility when they have chronic illnesses such as HF. They often move from one facility to another (including alternative healthcare facilities) seeking a cure.

The relatively low mortality rate in our cohort may be related to the fact that the study was conducted in a cardiology unit and may not reflect what happens in a general medical ward or in private practice in the country. The clinical characteristics of our patients may also be explanatory. Our subjects were younger compared to the typical patients with HF in the Western world, who are generally elderly.

The average length of hospital stay was longer in our setting (nine days) compared to 6.1 days in the USA^28^ and nine days in Europe.^7^ However it was shorter than the 21 days reported from Japan.^37^ It is possible that longer stay in hospital affords patients the opportunity to recover well and get used to medications for HF. HF outcome is generally better in Japanese patients compared to other high-income countries.^7^,^8^,^10^

Furthermore it is also possible that the aetiology of HF in our cohort could have affected the outcome. Hypertension is predominantly the major risk factor for HF in our cohort. Ischaemic heart disease is relatively uncommon. It is well known that mortality rates from coronary artery disease (CAD) are generally worse than in those with non-ischaemic heart disease. Mitchell *et al.*^38^ reported a total mortality rate of 30% at three years in the placebo group of ischaemic HF patients compared to a rate of 15% in the non-ischaemic HF group.

The poorer outcome of women in our study may be because the women were less educated and more likely to be unemployed and dependent than the men, and may not be able to pay for HF medications. Clinic follow up may also be poorer in the women.

The finding of low frequency of use of some disease-modifying drugs in our cohort is an opportunity for future intervention in HF management in our environment. This is because studies have shown that ACE inhibitors,^39^ ARBs,^40^ and beta-blockers^12^ can improve survival in patients with HF. Furthermore, the African-American Heart Failure trial has shown the efficacy of the hydrallazine–isosorbide combination in the treatment of HF in blacks.^13^

The main aetiological factors for HF in our cohort were non-ischaemic in origin, with hypertensive heart disease being responsible for over 75% of cases. It may be reasonable to suggest that applying guidelines derived from clinical trials in the Western world, where most HF is ischaemic in origin, may be inappropriate in our population.

## Limitations

Our study was a single-centre, hospital-based study conducted in a cardiology unit and therefore may not have captured all the patients with heart failure in the city during the study period, although many referrals were received from surrounding hospitals and clinics during the period due to the awareness that was created of the study. The findings of the study may not be extrapolated to the general population or the situation in other Nigerian hospitals. A national HF registry is needed, as has been done in many other countries.

The use of the Framingham criteria as a screening tool may have missed some patients, especially the elderly with HF, as the criteria are not sensitive in this population.

Due to cost consideration, our subjects did not have NT-proBNP levels done as this has not become a routine practice in the country. NT-proBNP has been shown to be a strong predictor of prognosis in HF.^41^ Other prognostic variables, such as exercise capacity (VO_2_ and six-minute walk) were also not assessed in our patients.

Some of our patients were lost to follow up and this may have affected the survival information in this study. However the rate of attrition was similar to that in other follow-up studies.^8^,^11^ This was complicated by the fact that there is no effective national death registry in the country. We also could not ascertain the exact cause of death for patients who died outside the hospital environment.

## Conclusions

The characteristics of the HF population in Nigeria are different from similar populations in high-income countries. Our patients are about 20 years younger and have non-ischaemic aetiological risk factors for HF, especially hypertensive heart disease. Short-or medium-term outcome is relatively lower than (or comparable to) findings from high-income countries and have some similar prognostic factors, such as renal function, anaemia, body mass index, blood pressure parameters, as well as ECG and echocardiographic variables. There is a need for a national HF registry in the country to better understand the characteristics, management and outcome of HF in the different regions of the country.
